# Matching‐adjusted indirect comparison of efficacy and safety of bortezomib, thalidomide, and dexamethasone (VTd) as per label compared with modified VTd dosing schedules in patients with newly diagnosed multiple myeloma who are transplant eligible

**DOI:** 10.1002/jha2.77

**Published:** 2020-08-25

**Authors:** Pieter Sonneveld, María‐Victoria Mateos, Adrian Alegre, Thierry Facon, Cyrille Hulin, Mahmoud Hashim, Talitha Vincken, Tobias Kampfenkel, Sarah Cote, Jianming He, Annette Lam, Philippe Moreau

**Affiliations:** ^1^ Erasmus MC Cancer Institute Rotterdam Netherlands; ^2^ University Hospital of Salamanca/IBSAL Salamanca Spain; ^3^ Hospital Universitario de La Princesa Madrid Spain; ^4^ University of Lille, CHU Lille, Service des Maladies du Sang Lille France; ^5^ Hospital Center University De Bordeaux Bordeaux France; ^6^ Ingress Health Rotterdam Netherlands; ^7^ Department of Oncology Janssen Research & Development, LLC Netherlands; ^8^ Janssen Global Services, LLC Raritan NJ USA; ^9^ Nantes University Hospital Hotel‐Dieu, Service Hematologie Nantes France

**Keywords:** bortezomib, CASSIOPEIA, multiple myeloma, thalidomide, VTd‐label, VTd‐mod

## Abstract

**Background:**

The combination of bortezomib, thalidomide, and dexamethasone (VTd) is a standard of care for transplant‐eligible patients with newly diagnosed multiple myeloma (NDMM). Although approved labeling for VTd includes an escalating thalidomide dose up to 200 mg daily (VTd‐label), a lower fixed dose of thalidomide (100 mg daily; VTd‐mod) has become commonplace in clinical practice. To date, no clinical trials comparing VTd‐mod with VTd‐label have been performed. Here, we compared outcomes for VTd‐mod with VTd‐label using a matching‐adjusted indirect comparison.

**Methods:**

VTd‐mod data were from NCT02541383 (CASSIOPEIA; phase III) and NCT00531453 (phase II); VTd‐label data were from NCT00461747 (PETHEMA/GEM; phase III). To adjust for heterogeneity, baseline characteristics from VTd‐label were weighted to match VTd‐mod. Outcomes included overall survival (OS), progression‐free survival (PFS), postinduction and posttransplant responses, and safety.

**Results:**

VTd‐mod was noninferior to VTd‐label for OS, postinduction overall response rate (ORR), and very good partial response or better (≥VGPR). VTd‐mod was significantly better than VTd‐label for PFS, posttransplant ORR, and ≥VGPR. VTd‐mod was noninferior to VTd‐label for safety outcomes, and inferior to VTd‐label for postinduction and posttransplant complete response or better.

**Conclusions:**

Our analysis supports the continued use of VTd‐mod in clinical practice in transplant‐eligible NDMM patients.

## 1 INTRODUCTION

Treatment guidelines for patients with newly diagnosed multiple myeloma (NDMM) who are eligible for autologous stem cell transplantation (ASCT) recommend administration of a bortezomib‐based triple‐drug combination, including bortezomib, thalidomide, and dexamethasone (VTd), as a standard of care prior to transplantation [1]. The pretransplant induction regimen (referred to as VTd‐label; typically repeated for a maximum of six cycles) includes a 28‐day cycle composed of bortezomib (1.3 mg/m^2^ subcutaneously Days 1, 8, 15, and 22), escalating doses of thalidomide (100‐200 mg orally Days 1‐21), and dexamethasone (20 mg day of and day after bortezomib dosing, or 40 mg Days 1, 8, 15, and 22) [1, 2].

To mitigate the potentially toxic effects of high‐dose thalidomide exposure (such as thrombosis and peripheral neuropathy) [3], a reduced thalidomide dosing regimen (VTd‐mod; 100 mg daily) is commonly used in clinical practice. Despite routine clinical use, a clinical trial assessing efficacy and safety of VTd‐label versus VTd‐mod has not been performed. In the absence of head‐to‐head trials, indirect treatment comparisons may be done to assess the relative effectiveness of both regimens [4, 5]. Our objective was to use the matching‐adjusted indirect comparison (MAIC), which adjusts for heterogeneity in the baseline prognostic variables, to determine relative effectiveness of VTd‐mod versus VTd‐label treatment regimens in transplant‐eligible patients with NDMM [6, 7, 8].

## 2 METHODS

### 2.1 Data sources

Randomized controlled trials with a VTd treatment arm for treatment of transplant‐eligible patients with NDMM were identified via a systematic literature review; VTd dosing schedules were then evaluated to identify trials with VTd‐label and VTd‐mod arms. As such, other studies of VTd were excluded. The VTd‐label group comprised the VTd arm of the PETHEMA/GEM study (NCT00461747), a phase III randomized trial assessing three treatment regimens in patients with MM (alternating vincristine/carmustine/melphalan/cyclophosphamide/prednisone and vincristine/carmustine/doxorubicin/dexamethasone, followed by bortezomib, vs thalidomide/dexamethasone vs VTd) [8, 9]. The VTd regimen consisted of pre‐ASCT induction therapy (six 28‐day cycles) of bortezomib (1.3 mg/m^2^ Days 1, 4, 8, and 11), thalidomide (escalating doses: Cycle 1, 50 mg Days 1‐14; 100 mg Days 15‐28, and 200 mg thereafter), and dexamethasone (40 mg orally, Days 1‐4 and 9‐12). Post‐ASCT maintenance continued up to 3 years, and consisted of interferon α‐2b (3 MU subcutaneously, three times per week), thalidomide (orally, 100 mg daily), or thalidomide (orally, 100 mg daily) with bortezomib (one cycle every 3 months).

The VTd‐mod group consisted of pooled data from NCT02541383 (CASSIOPEIA; a phase III study) and NCT00531453 (a phase II study) [6, 7]. CASSIOPEIA was a two‐part, open‐label study conducted in transplant‐eligible patients with NDMM [7]. Part 1 consisted of patients randomized 1:1 to pre‐ASCT induction therapy (four 28‐day cycles, Cycles 1‐4) with either VTd‐mod or VTd‐mod with daratumumab (D‐VTd), an anti‐CD38 monoclonal antibody with immunomodulatory effects, followed by post‐ASCT consolidation therapy (two 28‐day cycles) of VTd‐mod or D‐VTd (daratumumab 16 mg/kg intravenously [IV] once weekly Cycles 1‐2; every 2 weeks Cycles 3‐6; bortezomib 1.3 mg/m^2^ [subcutaneously Days 1, 4, 8, and 11]; thalidomide 100 mg/day [orally]; and dexamethasone [orally or IV; Cycles 1‐2: 40 mg Days 1, 2, 8, 9, 15, 16, 22, and 23; Cycles 3‐4: 40 mg Days 1 and 2, then 20 mg Days 8, 9, 15, and 16]; Cycles 5‐6: 20 mg Days 1, 2, 8, 9, 15, and 16). Part 2 of CASSIOPEIA is ongoing, with re‐randomization of patients achieving a partial response or better at Day 100 posttransplant to observation or daratumumab maintenance (16 mg/kg, monotherapy) every 8 weeks until disease progression or for a maximum of 2 years. In the randomized NCT00531453 trial, NDMM patients received VTd‐mod composed of bortezomib (1.3 mg/m^2^ Days 1, 4, 8, and 11), dexamethasone (40 mg Days 1‐4 and 9‐12), and thalidomide (100 mg Days 1‐21) for four 21‐day cycles, followed by ASCT [6].

For the CASSIOPEIA (VTd‐mod) and PETHEMA/GEM (VTd‐label) trials, individual patient‐level data (IPD) were obtained from the sponsor and were validated with their respective clinical study reports. For the NCT00531453 study, only aggregate data were available, with all available baseline variables and outcomes extracted from primary publications; IPD were reconstructed from Kaplan‐Meier survival curves using a validated method [10].

### 2.2 Matching‐adjusted indirect comparison

Both naïve comparisons (without any adjustments) and MAICs (unanchored indirect comparison) were conducted; the efficacy and safety populations from CASSIOPEIA were used for comparison of efficacy and safety endpoints in their respective MAICs. For the MAIC, individual patients in the VTd‐label group were weighted based on baseline characteristics to match VTd‐mod. Prognostic factors considered for matching were selected in collaboration with clinical experts, and included all commonly available baseline patient demographic and clinical characteristics in both studies: age, sex, Eastern Cooperative Oncology Group (ECOG) performance status, myeloma type, International Staging System (ISS), creatinine clearance, hemoglobin level, and platelet count. Cytogenetic risk was not reported for NCT00531453; therefore, it was not possible to consider for matching.

### 2.3 Analysis endpoints and statistical methodology

Efficacy outcomes included overall survival (OS), progression‐free survival (PFS), and postinduction and posttransplant responses: overall response rate (ORR), complete response or better (≥CR), and very good partial response or better (≥VGPR). Safety outcomes included treatment discontinuation due to any grade adverse events (AEs), Grade 3 or 4 thrombosis, and Grade 3 or 4 peripheral neuropathy.

Comparison of VTd‐mod with VTd‐label was performed stepwise via a naïve, unadjusted, indirect comparison followed by MAIC. Baseline characteristics from patients in the VTd‐label arm from the PETHEMA/GEM study were utilized to weight IPD in the VTd‐label group and then matched with those in the pooled VTd‐mod group. Hazard ratios (HRs) with two‐sided 95% confidence intervals (CIs) for time to event outcomes (OS and PFS) were determined using a weighted Cox regression analysis model that was fitted to the treatment arm; a log‐rank test was used to determine *P*‐values for HRs and Kaplan‐Meier curves. Absolute rate difference and odds ratios (ORs), calculated with two‐sided 95% CIs, were used to determine treatment response and safety outcomes; *P*‐values for ORs were determined using the two‐sided Fisher's exact test. Based on recent oncology studies, results that did not achieve statistical significance (5%) were interpreted with the use of noninferiority margins [11, 12]. Noninferiority margins for response, safety, PFS, and OS were identified as 13% (rate difference), 13% (rate difference), 1.333 (HR), and 1.298 (HR), respectively [11].

## 3 RESULTS

Median duration of follow‐up was 35.9 months for VTd‐label, 33.3 months for NCT00531453 (VTd‐mod), and 18.8 months for CASSIOPEIA (VTd‐mod).

### 3.1 Efficacy

Baseline characteristics used for matching the VTd‐mod (n = 591; NCT00531453 [n = 49] + CASSIOPEIA [n = 542]) and VTd‐label (n = 130; PETHEMA/GEM) groups in the efficacy analyses are shown in Table  1. Prior to weighting, imbalances between groups were observed for ECOG status, myeloma type, ISS, and creatinine clearance; however, after weighting, baseline characteristics were similar across groups, and the cohorts were considered viable for comparison of efficacy.

**TABLE 1:** Baseline characteristics of the pooled VTd‐mod and VTd‐label pre‐ and postweighting arms for the efficacy analysis

Variable	VTd‐mod pooled (CASSIOPEIA; NCT00531453 [2013 publication])	VTd‐label before weighting (PETHEMA/GEM)	VTd‐label postweighting (PETHEMA/GEM)
Sample size	591	130	NA
Effective sample size	NA	NA	105
Median age (years)	58	57	58
Male (%)	58	58	58
Patients with ECOG ≥ 1 (%)	52	56	52
Patients with IgG myeloma (%)	61	66	61
ISS stage (%)			
I	41^*^	34	41
II	43	44	43
Creatinine clearance (median [mL/min])	95.2^*^	82.5	95.2

Abbreviations: ECOG, Eastern Cooperative Oncology Group; IgG, immunoglobulin G; ISS, International Staging System; NA, not applicable; VTd‐label, bortezomib, thalidomide, and dexamethasone as per label; VTd‐mod, modified bortezomib, thalidomide, and dexamethasone dosing.

* Relative difference of 10% or more compared to the same variable in PETHEMA/GEM.

VTd‐label: 100‐200 mg; VTd‐mod: fixed 100‐mg dose.

### 3.2 OS and PFS comparisons

**FIGURE 1:**
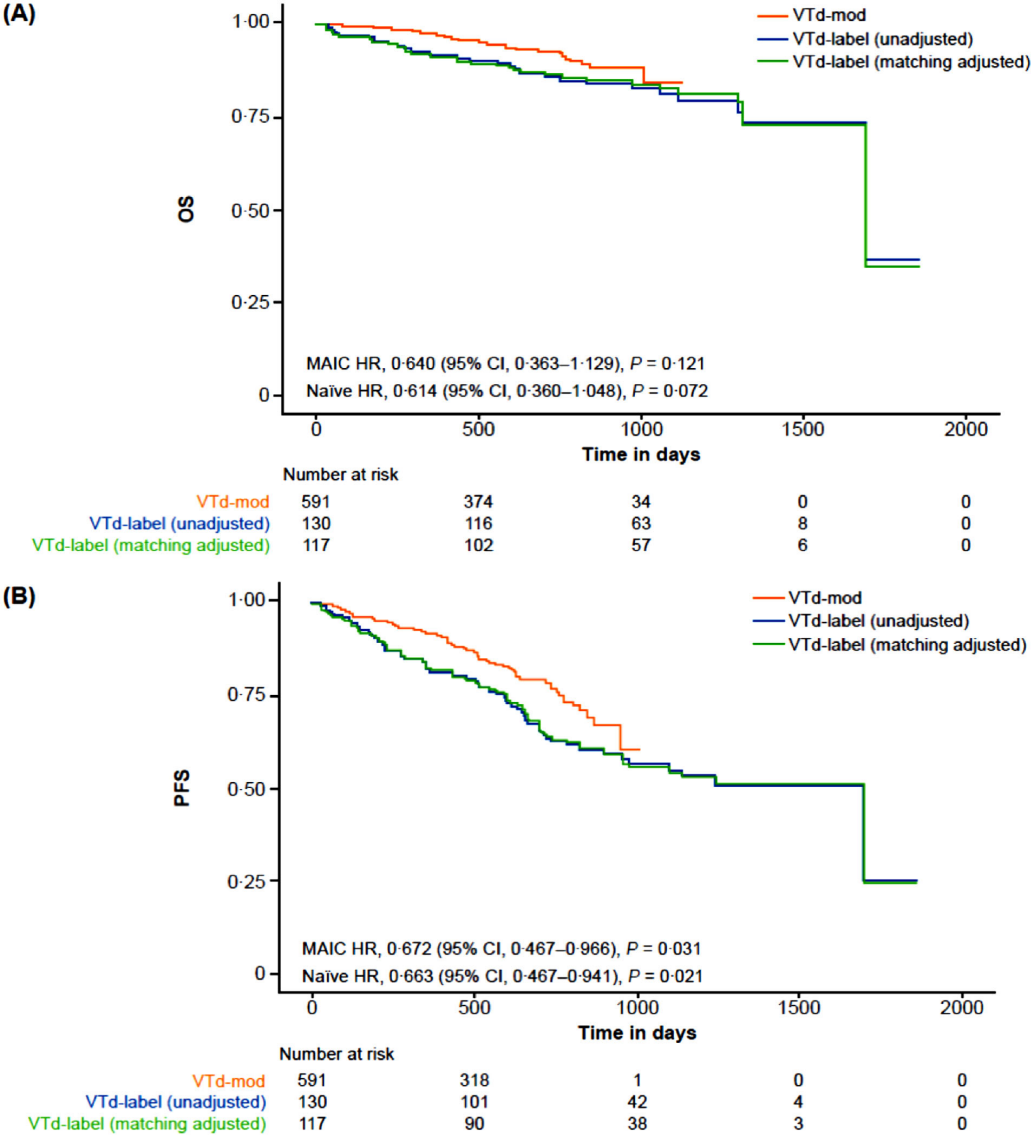
Comparison of VTd‐mod and VTd‐label for OS and PFS. Kaplan‐Meier estimates of OS (A) and PFS (B) in patients from the pooled NCT00531453 and CASSIOPEIA trials who received VTd‐mod versus patients from the PETHEMA/GEM trial who received VTd‐label *Note*. A weighted Cox regression model was used to estimate HR with two‐sided 95% CI in time‐to‐event outcomes. Noninferiority margins for OS and PFS were established as 1.333 and 1.298, respectively. VTd‐label: 100‐200 mg thalidomide. VTd‐mod: fixed 100‐mg thalidomide dose. CI, confidence interval; HR, hazard ratio; MAIC, matching‐adjusted indirect comparison; OS, overall survival; PFS, progression‐free survival; VTd‐label, bortezomib, thalidomide, and dexamethasone as per label; VTd‐mod, modified bortezomib, thalidomide, and dexamethasone dosing.

For OS, VTd‐mod was noninferior to VTd‐label for both the naïve comparison prior to weighted matching (HR = 0.614; 95% CI, 0.360‐1.048; *P* = .072) and on the MAIC (HR = 0.640; 95% CI, 0.363‐1.129; *P* = .121) (Figure 1A). Significant improvements in PFS were observed with VTd‐mod compared with VTd‐label for both naïve comparisons (HR = 0.663; 95% CI, 0.467‐0.941; *P* = .021) and the MAIC (HR = 0.672; 95% CI, 0.467‐0.966; *P* = .031) (Figure 1B).

### 3.3 Responses postinduction and posttransplant

Table 2 presents comparisons of postinduction and posttransplant treatment responses. For naïve, unadjusted comparisons, VTd‐mod was inferior to VTd‐label for postinduction and posttransplant ≥CR. VTd‐mod was noninferior to VTd‐label for postinduction ORR and ≥VGPR, but superior to VTd‐label for posttransplant ORR and ≥VGPR.

**TABLE 2:** Postinduction and posttransplant response rates from the naïve comparisons and the MAIC analyses of pooled VTd‐mod versus VTd‐label

Outcome/analysis	VTd‐label n (%)	VTd‐mod pooled n (%)	Rate difference (95% CI)	Odds ratio (95% CI)	*P*‐value^*^	Superior/noninferior/inferior
Response postinduction						
≥CR						
Naïve	46 (35.4)	50 (8.5)	–26.92 (–35.44 to ‐18.4)	0.169 (0.106‐0.268)	<.0001	Inferior
MAIC	42 (36.0)	50 (8.5)	–27.51 (–36.5 to –18.52)	0.165 (0.102‐0.265)	<.0001	Inferior
≥VGPR						
Naïve	64 (49.2)	338 (57.2)	7.96 (–1.51 to 17.44)	1.378 (0.942‐2.016)	.118	Noninferior
MAIC	63 (54.0)	338 (57.2)	3.18 (–6.7 to 13.07)	1.138 (0.764‐1.695)	.541	Noninferior
ORR						
Naïve	110 (84.6)	536 (90.7)	6.08 (–0.55 to 12.71)	1.772 (1.021‐3.075)	.055	Noninferior
MAIC	99 (84.5)	536 (90.7)	6.15 (–0.82 to 13.11)	1.781 (1.004‐3.16)	.065	Noninferior
Response posttransplant						
≥CR						
Naïve	61 (46.9)	94 (15.9)	–31.02 (–40.09 to –21.95)	0.214 (0.142‐0.322)	<.0001	Inferior
MAIC	55 (47.4)	94 (15.9)	–31.52 (–41.04 to –21.99)	0.210 (0.137‐0.321)	<.0001	Inferior
≥VGPR						
Naïve	72 (55.4)	406 (68.7)	13.31 (3.99 to 22.64)	1.768 (1.200‐2.603)	.004	Superior
MAIC	67 (57.7)	406 (68.7)	10.95 (1.24 to 20.66)	1.606 (1.070‐2.41)	.0024	Superior
ORR						
Naïve	101 (77.7)	536 (90.7)	13.00 (5.47 to 20.53)	2.798 (1.701‐4.602)	<.0001	Superior
MAIC	927 (78.6)	536 (90.7)	12.14 (4.34 to 19.95)	2.661 (1.579‐4.484)	.001	Superior

Abbreviations: ≥CR, complete response or better; ≥VGPR, very good partial response or better; CI, confidence interval; MAIC, matching‐adjusted indirect comparison; ORR, overall response rate; VTd‐label, bortezomib, thalidomide, and dexamethasone as per label; VTd‐mod, modified bortezomib, thalidomide, and dexamethasone dosing.

* Two‐sided *P*‐values based on Fisher's exact test. VTd‐label: 100‐200 mg; VTd‐mod: fixed 100‐mg dose.

When comparing postinduction responses on matching‐adjusted samples, VTd‐mod was noninferior to VTd‐label, with similar ORR (90.7% vs 84.5%, respectively; rate difference, 6.15 [95% CI, –0.82 to 13.11]; *P* = .065) and ≥VGPR (57.2% vs 54.0%, respectively; rate difference, 3.18 [95% CI, –6.7 to 13.07]; *P* = .541) (Table 2).

For posttransplant responses after matching, VTd‐mod was superior to VTd‐label, with significantly higher ORR (90.7% vs 78.6%, respectively; rate difference, 12.14 [95% CI, 4.34‐19.95]; *P* = .001) or ≥VGPR (68.7% vs 57.7%, respectively; rate difference, 10.95 [95% CI, 1.24‐20.66]; *P *= .0024) (Table 2).

VTd‐mod was inferior to VTd‐label for ≥CR postinduction (8.5% vs 36.0%, respectively; rate difference, –27.51 [95% CI, –36.5 to –18.52]; *P *< .0001) and posttransplant (15.9% vs 47.4%, respectively; rate difference, –31.52 [95% CI, –41.04 to –21.99]; *P *< .0001) (Table 2).

### 3.4 Safety

Baseline characteristics from 587 pooled VTd‐mod patients (CASSIOPEIA: n = 538; NCT00531453: n = 49) and 130 VTd‐label patients before and after weighting were presented for MAIC safety analysis. Characteristics were similar after weighting (Table  3).

**TABLE 3:** Baseline characteristics of the pooled VTd‐mod and VTd‐label used for matching in the MAIC safety analysis

Variables	VTd‐mod pooled (CASSIOPEIA; NCT00531453 [2013 publication])	VTd‐label (PETHEMA/GEM)	VTd‐label postweighting (PETHEMA/GEM)
Sample size	587	130	NA
Effective sample size	NA	NA	116
Median age (years)	58	57	58
Male (%)^a^	58	58	58
Patients with ECOG ≥1 (%)^b^	52	56	52
Patients with IgG myeloma (%)^c^	61	66	61
ISS stage (%)^d^			
I	41^*^	34	41^*^
II	43	44	43
Creatinine clearance (median [mL/min])	95.2^*^	82.5	95.2^*^

Abbreviations: ECOG, Eastern Cooperative Oncology Group; IgG, immunoglobulin G; ISS, International Staging System; MAIC, matching‐adjusted indirect comparison; NA, not applicable; VTd‐label, bortezomib, thalidomide, and dexamethasone as per label; VTd‐mod, modified bortezomib, thalidomide, and dexamethasone dosing.

a Reference category for this variable was female sex.

b Reference category for this variable was ECOG > 1.

c Reference category for this variable was non‐IgG myeloma type.

d ISS staging is a variable with three levels: ISS I, ISS II, and ISS III. This variable was dummy coded into two variables (ISS I and ISS II) with ISS III used as the reference category.

MAIC analysis of safety outcomes revealed VTd‐mod was noninferior to VTd‐label. No statistically significant differences between VTd‐mod and VTd‐label were found for the rate of discontinuation due to AEs for either the naïve comparison (5.5% vs 6.2%; rate difference, −0.70 [95% CI, –5.22 to 3.82]; *P* = .678) or MAIC (5.5% vs 5.6%; rate difference, −0.18 [95% CI, −4.65 to 4.29]; *P* = .831) (Table  4). Incidence of Grade 3 or 4 thrombosis based on naïve comparison was 2.0% for VTd‐mod versus 0.8% for VTd‐label (rate difference, 1.28 [95% CI, −0.61 to 3.16]; *P* = .481) and 2.0% for VTd‐mod versus 0.9% for VTd‐label (rate difference, 1.11 [95% CI, −0.95 to 3.16]; *P* = .709) for MAIC. Rates of Grade 3 or 4 peripheral neuropathy were 6.6% versus 5.4% (naïve comparison; rate difference, 1.26 [95% CI, −3.11 to 5.63]; *P* = .696) and 6.6% versus 4.2% (MAIC; rate difference, 2.40 [95% CI, −1.70 to 6.49]; *P* = .409).

**TABLE 4:** AEs summary for pooled VTd‐mod versus VTd‐label

	Analysis	VTd‐mod, n (%)	VTd‐label, n (%)	Rate difference, % (95% CI)	*P*‐value^*^	Superior/Noninferior/Inferior
Discontinuation due to AEs	Naïve	32 (5.5)	8 (6.2)	–0.70 (–5.22 to 3.82)	.678	Noninferior
	MAIC	32 (5.5)	7 (5.6)	–0.18 (–4.65 to 4.29)	.831	Noninferior
Grade 3 or 4 thrombosis	Naïve	12 (2.0)	1 (0.8)	1.28 (–0.61 to 3.16)	.481	Noninferior
	MAIC	12 (2.0)	1 (0.9)	1.11 (–0.95 to 3.16)	.709	Noninferior
Grade 3 or 4 peripheral neuropathy	Naïve	39 (6.6)	7 (5.4)	1.26 (–3.11 to 5.63)	.696	Noninferior
	MAIC	39 (6.6)	5 (4.2)	2.40 (–1.70 to 6.49)	.409	Noninferior

Abbreviations: AEs, adverse events; CI, confidence interval; VTd‐label, bortezomib, thalidomide, and dexamethasone as per label; VTd‐mod, modified bortezomib, thalidomide, and dexamethasone dosing.

* Two‐sided *P*‐value based on Fisher's exact test.

## 4 DISCUSSION

The triple‐drug combination regimen, VTd, is regarded in clinical practice as a standard of care for patients with NDMM who are eligible for transplant [1, 13, 14]. Higher doses of thalidomide have been associated with AEs such as peripheral neuropathy, so clinicians have introduced a lower, fixed‐dose regimen (100 mg) to offset these toxic effects [3, 7, 15, 16]. This lower‐dose regimen was administered to NDMM patients before and after ASCT in the NCT00531453 study and the phase III CASSIOPEIA study [6, 7], but no direct comparisons between the higher‐ and lower‐dose thalidomide regimens have been conducted in randomized, controlled clinical trials. In order to compare the efficacy and safety of these regimens in the absence of a direct, comparative trial, indirect analysis methods such as MAIC may be employed, which apply weighting factors to alleviate heterogeneity within analysis groups that may adversely skew interpretation of outcomes and hinder clinical decision‐making [4, 5]. Here, MAIC estimated efficacy and safety for VTd‐mod versus VTd‐label by comparing outcomes.

The MAIC analysis demonstrated that VTd‐mod was noninferior to VTd‐label for OS, postinduction ≥VGPR and ORR, and safety endpoints, inferior to VTd‐label for postinduction and posttransplant ≥CR, and superior to VTd‐label on PFS and posttransplant ≥VGPR and ORR. The efficacy results, with VTd‐mod showing noninferior or better efficacy on some endpoints, but inferior efficacy on others, were unexpected. It is possible that study results were affected by patient selection bias, because the studies were performed at different times and in different geographical locations. Different reporting rules and standards could also have affected the results. For example, response criteria for CASSIOPEIA and NCT00531453 were more rigorous than those in PETHEMA/GEM. Response in CASSIOPEIA and NCT00531453 was determined using restrictive criteria from the International Myeloma Working Group (and in CASSIOPEIA, a strict computer algorithm was also used), whereas response in PETHEMA/GEM was investigator assessed using criteria from the European Society for Blood and Marrow Transplantation [6, 7, 8]. The stricter criteria used to assess response in the VTd‐mod studies may have resulted in an underestimation of the benefit of VTd‐mod versus VTd‐label, which may explain the inferiority for VTd‐mod over VTd‐label on some efficacy endpoints.

Differences in study design, such as follow‐up times, routes of treatment administration, and maintenance regimens, may also have introduced bias toward the VTd‐label group, specifically regarding long‐term survival. The median follow‐up time in PETHEMA/GEM for VTd‐label was longer (35.2 months) [8, 9] than that in CASSIOPEIA (18.8 months) [7]. In addition, per design of CASSIOPEIA, patients with at least a partial response were re‐randomized (100 days post‐ASCT) to either observation or daratumumab monotherapy (every 8 weeks) for a further 2 years. Thus, fewer patients in CASSIOPEIA received maintenance treatment, so this analysis likely underestimates the survival benefit of VTd‐mod versus VTd‐label and should be considered conservative.

In terms of safety, the lack of superiority of VTd‐mod compared with VTd‐label was also unexpected, but reporting rules for PETHEMA/GEM may not have been as rigorous as those for CASSIOPEIA, thereby underestimating some AEs. As VTd‐mod is often used in clinical practice to reduce AEs, such as peripheral neuropathy, and thereby prevent early treatment discontinuations, analysis confirming reduced toxicity would be beneficial for clinical and reimbursement decision‐making. Although this MAIC analysis cannot confirm a lower rate of safety events based on included endpoints, it can confirm that VTd‐mod is noninferior to VTd‐label, supporting the use in NDMM patients.

Because our MAIC analysis was a cross‐trial comparison, statistical adjustments compensating for unreported or unobserved confounding factors, referred to as residual confounding, could not be performed and may introduce bias. Residual confounding may result in omission of variables critical to balancing patient groups [17]. The included trials collected baseline data that were relevant to clinical practice, thereby reducing the risk of residual confounding; however, matching cannot adjust for the differences in patients in terms of their standard of care at different time periods. Although this analysis stems from randomized clinical trials, it assumes that absolute outcomes can be predicted from the covariates (baseline variables) [5]. This assumption is strong and difficult to meet. Nevertheless, in the absence of IPD being available for all the studies included, MAIC is the best method to derive indirect evidence between two treatment regimens. There are also limitations concerning the method used to extract IPD. The NCT00531453 study data were derived from aggregate data extracted from primary publications, so Kaplan‐Meier curves generated to reproduced IPD may have been negatively impacted by variability of image quality and censoring of data. Finally, MAIC analysis utilizes weighting to minimize heterogeneity within patient populations; however, such methodology necessarily reduces the sample size, consequently negatively impacting precision of estimates.

## 5 CONCLUSIONS

Based on this MAIC analysis, VTd‐mod was shown to be noninferior to VTd‐label for OS, postinduction ≥VGPR, and ORR. This analysis also demonstrated the superiority of VTd‐mod for PFS, and posttransplant ≥VGPR and ORR. For response postinduction and posttransplant, VTd‐mod was inferior to VTd‐label for ≥CR. Although VTd‐mod patients received a lower thalidomide dose compared with VTd‐label patients, safety outcomes, even for Grade 3 or 4 events, were non‐inferior but did not reach the level of superiority. Taken together, our analysis supports the continued use of VTd‐mod in clinical practice in transplant‐eligible NDMM patients. These findings may also provide useful insight for clinical and reimbursement decision‐making regarding the relative efficacy and safety of the different treatment regimens.

## AUTHOR CONTRIBUTIONS

PS, M‐VM, AA, TF, CH, and PM contributed to the accrual and treatment of patients, data acquisition, and interpretation and analysis of data from the CASSIOPEIA study. TK, SC, JH, and AL contributed to the study concept, designed the MAIC analysis, and interpreted the data. MH and TV performed the MAIC analyses. All authors participated in manuscript preparation and revisions, and all authors read and approved the final manuscript.

## CONFLICT OF INTERESTS

PS: Honoraria from Amgen, BMS, Celgene, Janssen, Karyopharm; Takeda; research funding from Amgen, Celgene, Janssen, Karyopharm, SkylineDx, Takeda. M‐VM: advisory committee for AbbVie, Amgen, Celgene, Genentech, GSK, Janssen, Mundipharma EDO, PharmaMar, Roche Laboratories, Inc, Takeda; membership on an entity's board of directors for AbbVie, Amgen, Celgene, EDO, GSK, Janssen, PharmaMar, Takeda; honoraria from Adaptive, Amgen, Celgene, Janssen, Takeda; speakers bureau for Amgen, Celgene, Janssen, Takeda; data and monitoring committee for Amgen and Jansen. AA: Advisory committee for Amgen, Celgene, Janssen, Sanofi, Takeda; consultancy for Sanofi; honoraria from Amgen, Celgene, Janssen; research funding from Celgene, Janssen, Sanofi. TF: Membership on an entity's board of directors or advisory committees for Amgen, Celgene, Janssen, Karyopharm, Oncopeptides, Roche, Sanofi, Takeda; speakers bureau for Celgene, Janssen, Takeda. CH: Consultancy for Celgene; honoraria from AbbVie, Amgen, Celgene, Janssen. MH and TV: Employment from Ingress‐Health. TK, SC, JH, and AL: Employment from Janssen. PM: Consultancy for AbbVie, Amgen, Celgene, Janssen, Takeda; honoraria from AbbVie, Amgen, Celgene, Janssen, Takeda.

## CLINICAL TRIAL REGISTRATION

NCT00531453 (phase II), CASSIOPEIA study (NCT02541383), and PETHEMA/GEM study (NCT00461747).

## DATA SHARING AGREEMENT

The data sharing policy of Janssen Pharmaceutical Companies of Johnson & Johnson is available at https://www.janssen.com/clinical-trials/transparency. As noted on this site, requests for access to the study data can be submitted through Yale Open Data Access (YODA) Project site at http://yoda.yale.edu.
